# Microglia and Their Promising Role in Ischemic Brain Injuries: An Update

**DOI:** 10.3389/fncel.2020.00211

**Published:** 2020-07-10

**Authors:** Luting Yu, Xiaojuan Su, Shiping Li, Fengyan Zhao, Dezhi Mu, Yi Qu

**Affiliations:** ^1^Department of Paediatrics, West China Second University Hospital, Sichuan University, Chengdu, China; ^2^Key Laboratory of Birth Defects and Related Diseases of Women and Children, Sichuan University, Ministry of Education, Chengdu, China

**Keywords:** ischemic brain injuries, microglia, polarization, depletion, repopulation

## Abstract

Ischemic brain injuries are common diseases with high morbidity, disability, and mortality rates, which have significant impacts on human health and life. Microglia are resident cells of the central nervous system (CNS). The inflammatory responses mediated by microglia play an important role in the occurrence and development of ischemic brain injuries. This article summarizes the activation, polarization, depletion, and repopulation of microglia after ischemic brain injuries, proposing new treatment strategies for such injuries through the modulation of microglial function.

## Introduction

Ischemic brain injuries mainly contain ischemic stroke, cerebral white-matter ischemia, and neonatal hypoxia-ischemic brain damage, which are the third-leading cause of human disability, and the second-leading cause of death in the world (Desmond et al., 2002; Candelario-Jalil, 2009; Mozaffarian et al., 2016). Epidemiological data indicate that approximately 17 million people suffer from ischemic brain injuries each year (Brainin et al., 2015; Béjot et al., 2016). Inflammatory responses play important roles in the pathogenesis of ischemic brain injuries, and inhibition of these responses improves the neurological deficits in cerebral-ischemic animal models (Wang, 2005; Yilmaz and Granger, 2008). Microglia are resident macrophage-like immune cells in the central nervous system (CNS) and are considered sentinels at the forefront of the neuroinflammatory response to different brain insults (Lawson et al., 1990; Eldahshan et al., 2019). Recent studies have shown that microglia can be divided into two polarizing phenotypes: the pro-inflammatory and neurotoxic M1, and the anti-inflammatory and neural-repair M2 phenotypes (Franco and Fernández-Suárez, 2015). The transition of these microglial phenotypes is closely related to the degree of brain damage, which determines the prognosis after ischemic brain injuries (Kanazawa et al., 2017; Jiang et al., 2020). This article summarizes the roles of microglia in the pathogenesis of ischemic brain injuries and proposes new treatment strategies through the modulation of microglial function.

## Microglia Development and Function

Microglia are widely distributed in the CNS. They are highly specialized and dynamic resident macrophage-like cells of the brain, accounting for about 10% of the total number of CNS cells (Masuda and Prinz, 2016; Wolf et al., 2017; Zhang et al., 2018). The origin of microglia is controversial. For a long time, most scholars believed that the CNS microglia originated from bone marrow hematopoietic stem cells of the mesoderm, which differentiate into monocytes and then enter the brain through the circulatory system (Chan et al., 2007). However, recent evidence suggests that the adult microglia are derived from primitive myeloid precursor cells in the embryonic day 8.0 (E8.0) yolk sac, which leave the yolk sac between E8.5 and E9.5, migrate to the developing neural tube through the primitive circulation, and finally differentiate into microglia (Ginhoux et al., 2010; Hoeffel et al., 2015). In human fetuses, microglia-like cells of varying morphologies can be detected at 3 weeks of gestation (Hutchins et al., 1990). By the mid-trimester, a limited number of amoeboid microglia are present (Choi, 1981; Fujimoto et al., 1989; Andjelkovic et al., 1998), and by 35 weeks of gestation, well-differentiated microglial populations with fully developed ramified morphologies can be found (Esiri et al., 1991; Verney et al., 2010). In mice, between gestational day (GD) 10 and 15, microglia are located near the capillaries of the CNS and are characterized by round or irregular-shaped cell bodies (Imamoto and Leblond, 1978). In rats, microglial influx occurs between GD 15 and 16, and branched microglia appear in the brain parenchyma between GD 18 and 19 (Kaur and Ling, 1991).

Microglia play an important role in the physiological processes of the CNS (Sevenich, 2018). When quiescent, microglia participate in modulating the special requirements of nerve-cell growth. They identify and remove pathogenic micro-organisms, allogeneic macromolecules, and allogeneic cells that invade the body (Ding et al., 2018). Furthermore, microglia regulate the number of neuronal precursor cells and participate in the formation and elimination of neuronal synapses (Frost and Schafer, 2016). In the normal brain, microglia are highly branched, continuously expanding and contracting protrusions at a high frequency. This behavioral characteristic of microglia provides the brain with a highly dynamic and efficient detection system to maintain CNS homeostasis (Parkhurst et al., 2013). Microglia are activated by infection, inflammation, trauma, or other injuries that occur in the brain. When activated, microglial bodies enlarge, become round or amoebic, and their synapses become shorter or disappear, to enhance their phagocytic and migratory abilities (Li et al., 2013).

## Activation, Proliferation, and Migration of Microglia After Ischemic Brain Injuries

Microglial activation can occur in the acute, sub-acute, and chronic phases of an ischemic stroke (Gulyas et al., 2012). During the acute phase, microglia respond to the overactivity of neurons within a few minutes (Eyo et al., 2014, 2015). Thirty minutes after the onset of middle cerebral artery occlusion (MCAO), activated microglia can be detected at the boundary of ischemic lesions. At 24 and 72 h, activated microglia can be found at the ischemic core and the boundary regions (Shi et al., 2015). During the chronic phase, activated microglia are located in the peri-infarct and distal regions initially. However, the number of amoeboid-like microglia would start to accumulate in the core area between 3 and 7 days after a stroke (Perego et al., 2011). In a model of cerebral white-matter ischemia, most of the microglia are activated after 3 days of injury, peaked at 10 days, and decreased after 1 month. This study showed cerebral hypoperfusion induced the activation and polarization of microglia, to enhance the production of pro-inflammatory cytokines (Qin et al., 2017).

Sapkota et al. (2017) used ionized calcium-binding adapter molecule 1 (Iba-1) and 5-Bromo-2′-deoxyuridine (BrdU) immuno-labeling to determine microglial proliferation process in the ischemic brain 3 days after MCAO. They found that the number of Iba-1/BrdU double-labeled cells were increased in the penumbra, indicating enhanced microglia proliferation in this area. Using positron-emission tomography and flow cytometry, Moraga et al. (2016) revealed that ischemia-induced proliferation of microglia in the ipsilateral cortex in an MCAO model. There is some evidence to suggest that the proliferation status of microglia is regulated by Toll-like receptor signaling. When using a Toll-like receptor 2 (TLR2) deficiency mice to establish the MCAO model, Bohacek et al. (2012) showed the number of proliferating microglia was lower in the brain of the TLR2 deficient mice 3 days after MCAO compared with the wild-type mice. Zhang et al. (2005) found that microglial proliferation was increased after the injection of TLR3 and TLR7/8 agonists in rats. Collectively, these studies suggest ischemic brain injuries can induce microglial proliferation, partly through the modulation of Toll-like receptor signaling.

Microglia can change their migratory ability when stimulated by local inflammations, shifting to the inflammatory lesions. Different receptors have different effects on microglial migration. It was found that G protein-coupled receptor 55 and 18 (GPR55 and GPR18) on the cell surface promote microglial migration (Kohno et al., 2006; Oka et al., 2007; McHugh et al., 2010; Kallendrusch et al., 2013), whereas mu-opioid receptors inhibited microglia migration (Chao et al., 1997). Lipopolysaccharides (LPS) exposure has been reported to inhibit microglial migration (Zhang et al., 2016). Although the exact mechanism remains elusive, there is evidence to suggest that LPS can attenuate microglia migration by down-regulating purinergic receptor P2Y12 (Charolidi et al., 2015). When activated, microglia migrate to the injury site, releasing not only inflammatory cytokines but also chemokines that recruit infiltrating leukocytes (Khan et al., 2017). These infiltrating leukocytes in turn release inflammatory cytotoxins, such as oxygen free-radicals and proteases to further damage cerebral tissues.

## Microglial Polarization After Ischemic Brain Injuries

**Figure 1:**
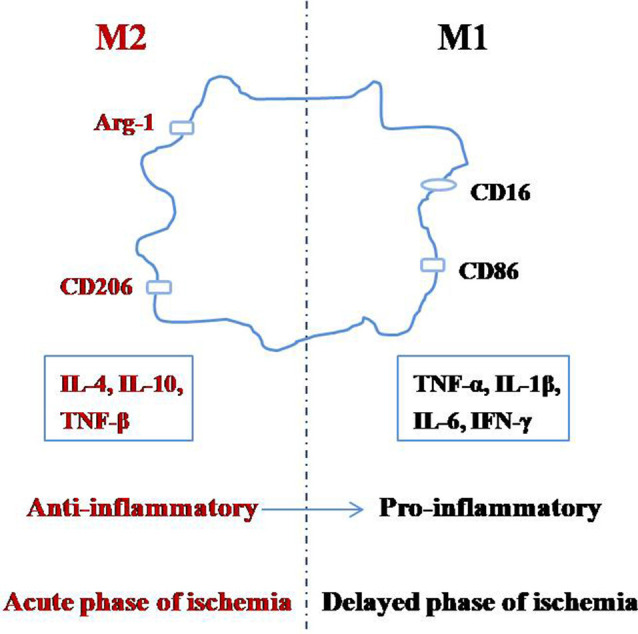
Polarization of microglia.

Microglia can switch between the M1 and M2 phenotypes depending on the changes in the environment by a process called polarization (Figure 1; Taylor and Sansing, 2013; Franco and Fernández-Suárez, 2015; Terashima et al., 2018; Zhang, 2019). The M1 phenotype, also known as the classical-activated type, primarily expresses surface antigens such as CD16 and CD86 (Shin et al., 2014). These microglia can secrete cytotoxic substances such as tumor necrosis factor-α (TNF-α), interleukin-1β (IL-1β), IL-6, interferon-γ (IFN-γ), other pro-inflammatory factors, and they promote the synthesis of reactive oxygen species and nitric oxide, which can be toxic to neurons and other glial cells (Zeng et al., 2018). The M2 phenotype is also known as the alternative-activation type, which mainly expresses surface antigens such as arginase-1 (Arg-1) and CD206 (Xiong et al., 2016). M2 microglia can engulf cell fragments or dead neurons, release anti-inflammatory factors such as IL-4, IL-10, and TNF-β to reduce inflammation and promote the survival of neurons (Kabba et al., 2018). Therefore, in addition to inhibiting microglial activation, adjusting the ratio of M2:M1 microglia is another important strategy to inhibit neuroinflammation.

After cerebral ischemia, the microglial polarization process is dynamic, with M2 microglia first appearing in the central region of ischemia, which gradually changes into the M1 phenotype (Hu et al., 2012; Li et al., 2018). It was reported that between 1 and 3 days after MCAO, M2 phenotype related genes are significantly increased, peaking between 3 and 5 days; while M1 phenotype related genes began to increase 3 days after MCAO and peaked at 14 days (Hu et al., 2012; Suenaga et al., 2015). These reports suggest that the early stages of ischemic brain injury are mainly M2 (anti-inflammatory) in nature, and only become M1 (pro-inflammatory) later on. This evidence supports the idea that the M1 microglia promote the release of inflammatory factors and aggravates brain injury, but M2 microglia reduce inflammation and protect the brain (Ma et al., 2019). The M2:M1 ratio can be increased by inhibiting M2 to M1 and promoting M1 to M2 transitions (Hashimoto et al., 2018), which can be achieved by inhibiting M1 signaling pathways, or adding M2 phenotype inducing factors to the microenvironment of the brain (Kanazawa et al., 2017).

The transitions between the M1 and M2 phenotypes are regulated by several factors. NF-κB mediates inflammatory responses and plays an important role in the activation of M1 microglia (Saijo and Glass, 2011; Liu et al., 2017). In an LPS-induced inflammation model, curcumin inhibited the formation of M1 microglia by reducing the activation of the TLR4/NF-κB pathway in the mouse microglial cell line BV2 cells (Zhang et al., 2019). Activation of the Notch signaling pathway enhanced microglial polarization to M1 and induced the expression of many inflammatory cytokines (Adler et al., 2003; Butovsky et al., 2007). Simvastatin can inhibit M1 and enhance M2 polarization by inhibiting Notch signaling (Wu et al., 2018). Mammalian target of rapamycin (mTOR) also plays an important role in the regulation of microglia polarization. Li et al. (2016) revealed that mTORC1 blockade can reduce the number of M1-phenotype microglia, decrease the production of pro-inflammatory cytokines and lesion size after a focal ischemic stroke.

In contrast to NF-κB, Notch, and the mTORC1 signaling pathways, cAMP-response element-binding protein (CREB) works in concert with CCAAT/enhancer-binding protein β (C/EBPβ) to enhance the expression of M2-specific genes. C/EBPβ has two opposing roles in the regulation of M1 and M2 phenotypes, which may be underlined by the fact that C/EBPβ can bind to both CREB and NF-κB, resulting in antagonistic downstream effects (Ruffell et al., 2009). Many members of the signal transducer and activator of transcription (STAT) family, including STAT1, STAT3, and STAT6, are involved in microglia phenotypic switching (Sica and Bronte, 2007). STAT1 can enhance microglia polarization to M1 when stimulated by IFN-γ (Qin et al., 2012a). STAT6 can promote microglia transition to the M2 phenotype when stimulated by IL-13 and IL-4 (Martinez-Nunez et al., 2011). The functions of STAT3 are variable, promoting microglia polarization to either the M1 or M2 phenotype when stimulated by IL-6 (Qin et al., 2012b) or IL-10 (Koscsó et al., 2013), respectively. Peroxisome proliferator-activated receptor-γ (PPAR-γ) is another transcription factor known to regulate microglial phenotype. PPAR-γ agonists (rosiglitazone and pioglitazone) have been shown to induce a microglial transition to theM2 phenotype in models of CNS disease associated with inflammation (Hasegawa-Moriyama et al., 2013). Similarly, activation of the AMP-activated protein kinase (AMPK) signaling pathway has also been shown to promote M2 macrophage/microglia polarization, thereby inhibits inflammation (Xu et al., 2015). Recent researches showed that microRNAs (miRNAs) play a role in microglia polarization. While miR-124, miR-155, and miR-689 are related to pro-inflammatory pathways and M1 polarization (Moore et al., 2013), miR-145 and miR-711 are associated with the anti-inflammatory pathways and M2-phenotype polarization (Ponomarev et al., 2011).

## Microglia as Therapeutic Targets for Ischemic Brain Injuries

### Regulation of Microglial Activation and Polarization

Many strategies have been developed to regulate microglia activation and polarization for the treatment of ischemic brain injuries (Table 1). After an ischemic stroke, metformin treatment has been shown to enhance cerebral AMPK activation, promote functional recovery, and shift microglia toward an M2 phenotype (Jin et al., 2014). Statins are lipid-lowering drugs that also have extensive anti-inflammatory and immune-modulatory effects (Greenwood et al., 2006). Studies have shown that atorvastatin could ameliorate functional recovery, reduced lesion volumes 24 h after an experimental stroke, reduce microglia activation, and decrease leucocyte adhesion and infiltration (Potey et al., 2015). Indomethacin, a non-steroidal anti-inflammatory drug, significantly reduced microglial activation in a rat model of focal ischemia model (Lopes et al., 2016). Noggin is an endogenous antagonist of bone morphogenetic proteins (BMPs) such as BMP 2 and BMP 4 (Shijo et al., 2018; Chien et al., 2020). In a rat model of amyotrophic lateral sclerosis, antagonizing BMP4 attenuated disease progression through suppressing microglia and astrocyte activation, and noggin supplementation significantly ameliorated the progression of the disease, suggesting that noggin might modulate glia function through regulating BMPs (Shijo et al., 2018). In the MCAO mice model, noggin was found to be involved in the regulation of microglia polarization. When noggin was injected into the ipsilateral ventricle of mice with MCAO, it reduced M1 markers (IL-1β, TNF-α, IL-12, and CD86) and increased M2 markers (IL-1α, IL-10, Arg-1, and CD206) in activated microglia, indicating that noggin can induce microglia to change from M1 to M2 phenotype (Shin et al., 2014). In a rat model of cerebral ischemia, treatment with resveratrol before ischemic injury reduced microglial activation, an effect that was attributed to the inhibition of NF-κB and Jun N-terminal kinase (JNK) activation (Simão et al., 2012). Lentivirus mediated IL-13 delivery promoted microglia differentiation to the M2 phenotype in a mouse model of multiple sclerosis, suggesting IL-13 gene immunotherapy is a potential treatment for neuroinflammation (Guglielmetti et al., 2016).

**Table 1:** Overview of drugs that regulate microglia activation and polarization.

Drug	Animal model	Microglia activation	M2/M1 ratio	Reference
Metformin	MCAO	Decrease	Increase	Jin et al. (2014)
Atorvastatin	MCAO	Decrease	Not shown	Potey et al. (2015)
Indomethacin	Striatal ischemia	Decrease	Not shown	Lopes et al. (2016)
Noggin	MCAO	Decrease	Increase	Shin et al. (2014)
Resveratrol	Cerebral ischemia	Decrease	Not shown	Simão et al. (2012)
IL-13	Multiple sclerosis	Decrease	Increase	Guglielmetti et al. (2016)
Salidroside	Cerebral ischemia	Decrease	Not shown	Liu et al. (2018)
Anisalcohol	LPS-stimulated BV2	Decrease	Increase	Xiang et al. (2018)
Malibatol	MCAO	Decrease	Increase	Pan et al. (2015)

*MCAO, middle cerebral artery occlusion; LPS, lipopolysaccharides*.

Some Chinese medicines have anti-inflammatory properties. Salidroside, an active ingredient isolated from a Chinese medicinal herb, Rhodiola sachalinensis, significantly reduced cerebral infarction, improved neurological function, reduced M1, but increased M2 microglia markers expression after an ischemic stroke (Liu et al., 2018). In LPS-stimulated BV2 microglia, anisalcohol inhibited M1 and promoted M2 transitions by inhibiting NF-κB and MAPK activation thus attenuated the production of inflammatory mediators (Xiang et al., 2018). In a mouse MCAO model, malibatol A, a natural anti-oxidant extract from plants, reduced the infarct size and alleviated brain damage. Its anti-inflammation effect was attributed to the reduction of M1 markers, and an increase of M2 markers in the microglia through the activation of nuclear receptor PPARγ (Pan et al., 2015).

### Regulation of Microglia Depletion and Repopulation

Several methods have been proposed to regulate microglia depletion and reproduction in the CNS (Table 2). Microglia survival and development are associated with colony-stimulating factor 1 receptor (CSF1R) signaling (Elmore et al., 2014, 2015) and studies have shown that microglia formation is blocked in CSF1R knockout mice (Ginhoux et al., 2010; Erblich et al., 2011). Spangenberg et al. (2016) found that inhibition of CSF1R could deplete microglia in the CNS. However, when CSF1R inhibition is removed, microglia rapidly repopulated the CNS (Elmore et al., 2014). In previous studies, CSF1R inhibition was able to deplete approximately 90% of microglia for 21 consecutive days, suggesting that microglial depletion might persist if CSF1R inhibition persisted (Szalay et al., 2016; Li et al., 2017). PLX3397 is a small molecule CSF1R inhibitor that can cross the blood-brain barrier (BBB), and could rapidly deplete microglia in the CNS (Elmore et al., 2014). When PLX3397 was used to treat mice for 7 days, more than 90% of the microglia in the brain was lost. After 21 days of treatment, more than 99% of all microglia in the brain were depleted (Elmore et al., 2014). This observation was confirmed by a similar study, in which 99% of microglia were eliminated by PLX3397 within 7 days (Najafi et al., 2018). Microglial repopulation began 48 h following drug withdrawal and the number of repopulating cells increased significantly after 72 h, with complete restoration of the microglia population in the CNS in 7 days (Elmore et al., 2014; Najafi et al., 2018). Other pharmacological approaches have also been used to selectively deplete microglia. PLX5662 is a CSF1R inhibitor with stronger BBB penetrating ability than PLX3397, which can lead to a depletion of Iba-1- and CD68-positive microglia in the CNS within 3 days (Acharya et al., 2016). Acute microglia depletion by PLX5662, and subsequent repopulation, can also resolve neuroinflammation and promote brain recovery (Rice et al., 2017). GW2580, another CSF1R inhibitor, can decrease the proliferating of microglia throughout the spinal cord (Gerber et al., 2018). However, CSF1R inhibition and subsequent microglial death during neurodevelopment may alter neuronal activity and synaptic physiology (Eyo and Wu, 2013). Therefore, future clinical applications of CSF1R inhibitors must consider these potential adverse effects.

**Table 2:** Overview of drugs that regulate microglia activation and polarization.

Drug	Animal model	Time window	Depletion efficiency	Repopulation	References
CSF1R inhibitor (PLX3397)	/	7 or 21 days	90% or 99%	Yes	Elmore et al. (2014)
CSF1R inhibitor (PLX3397)	Cerebral ischemia	21 days	97%	Not shown	Szalay et al. (2016)
CSF1R inhibitor (PLX3397)	/	7 days	99%	Yes	Najafi et al. (2018)
CSF1R inhibitor (PLX5562)	Radiation injury	3 days	95%	Not shown	Acharya et al. (2016)
CSF1R inhibitor (GW2580)	Spinal cord injury	10 weeks	not shown	Not shown	Gerber et al. (2018)
Liposomal clodronate	/	1 or 5 days	80%	Yes	Torres et al. (2016)
Mac-1-saporin	Spinal microglia depletion	1 day	50%	Yes	Yao et al. (2016)

In addition to CSF1R inhibitors, liposomal clodronate and Mac-1-saporin have also been shown to affect microglia depletion and repopulation (Torres et al., 2016; Yao et al., 2016). In a report by Torres et al. (2016), liposomal clodronate was injected into the hippocampus, and Iba-1 labeled microglia were analyzed between 1 and 7 days after injection. The study showed that microglia decreased significantly on day 1, which continued until day 5 and began to reappear on day 7 (Torres et al., 2016). On the other hand, depletion by Mac-1-saporin, a selective microglial immunotoxin, allowed microglia to repopulate rapidly (Yao et al., 2016).

Although several studies have shown that their population of microglia can fully restore microglial functions and attenuate brain damage (Rice et al., 2017; Zhang et al., 2018), the capacity for microglial repopulation is limited. While the depleted microglia repopulated 7 days after the termination of CSF1R inhibition, repopulation failed when the process was repeated (Najafi et al., 2018).

### Microglia Transplantation

Studies by Kitamura et al. (2004, 2005) have shown that after 1 h of cerebral ischemia, intracerebroventricular injection of microglia can improve neuronal survival, reduce neurodegeneration, and improve neurological behaviors in the transient MCAO mouse model. Using the same model, intravenous injection of microglia 48 h after cerebral ischemia reduced neuronal apoptosis, increased neurotrophic factors including glial cell line-derived neurotrophic factor (GDNF), brain-derived neurotrophic factor (BDNF), and anti-inflammatory cytokines IL-4 and IL-15, and promoted functional neurological recovery (Narantuya et al., 2010a). However, in the MCAO rat model, microglia transplantation via the tail vein did not affect neurological outcomes (Jiang et al., 2013). These conflicting results may be explained by the different experimental animals, time points, and the doses of microglia transplanted. In addition to the ischemic stroke model, microglia transplantation has also been used in models of chronic-ischemia, spinal cord injury, Alzheimer’s disease, and Parkinson’s disease, and have shown beneficial effects on tissue repair and functional recovery (Takata et al., 2007; Yu et al., 2009; Narantuya et al., 2010b; Danielyan et al., 2014).

## Conclusion

In summary, there is a close relationship between microglial function and the pathogenesis of ischemic brain injuries. The activation, polarization, depletion, and repopulation of microglia can determine the occurrence and the development of ischemic brain injuries. Polarization is a critical factor that modulates microglial-mediated inflammatory responses. M1 microglia can release pro-inflammatory cytokines, enhance local inflammatory cell infiltration, damage the BBB, induce neuronal death, thereby aggravating ischemic brain injuries. In contrast, M2 microglia can promote the expression of anti-inflammatory cytokines and neurotrophic factors, thereby alleviating ischemic brain injuries. Microglia polarization is regulated by some molecular signaling such as NF-κB, Notch, mTORC1, AMPK, STAT, and PPAR-γ. Accordingly, drugs that modulate these molecular signaling such as metformin, resveratrol, and anisalcohol were found to promote the transition from M1 to M2 phenotype of microglia. These drugs may provide potential benefits for the treatment of ischemic brain injuries. Other than the modulation of microglia polarization, regulation of depletion and repopulation is another newly established strategy to treat ischemic brain injuries. Some agents such as CSF1R inhibitors, Liposomal clodronate, and Mac-1-saporin were found to be potent in inducing microglia depletion and its subsequent repopulation, thereby attenuating inflammation after ischemic brain injuries. Furthermore, microglia transplantation can improve nervous system recovery under certain circumstances, which is worth exploring while developing therapies for ischemic brain injuries. With the deepening of researches in this field, more and more molecules and signaling pathways are involved in the regulation of microglial functions. As mentioned above, some miRNAs were found to play critical roles in the regulation of microglia polarization. Future works need to focus on the exploration of strategies that target these new-found molecules for the treatment of ischemic brain injuries.

## Author Contributions

LY, XS, SL, and FZ wrote the manuscript. DM proposed the idea for the manuscript. YQ was responsible for checking the whole manuscript.

## Conflict of Interest

The authors declare that the research was conducted in the absence of any commercial or financial relationships that could be construed as a potential conflict of interest.
